# A heart failure self-management program for patients of all literacy levels: A randomized, controlled trial [ISRCTN11535170]

**DOI:** 10.1186/1472-6963-6-30

**Published:** 2006-03-13

**Authors:** Darren A DeWalt, Robert M Malone, Mary E Bryant, Margaret C Kosnar, Kelly E Corr, Russell L Rothman, Carla A Sueta, Michael P Pignone

**Affiliations:** 1 Division of General Internal Medicine and Clinical Epidemiology, University of North Carolina School of Medicine, Chapel Hill, NC, USA; 2 Center for Health Services Research, Division of General Medicine, Vanderbilt University Medical Center, Nashville, TN, USA; 3 Division of Cardiology, University of North Carolina School of Medicine, Chapel Hill, NC, USA

## Abstract

**Background:**

Self-management programs for patients with heart failure can reduce hospitalizations and mortality. However, no programs have analyzed their usefulness for patients with low literacy. We compared the efficacy of a heart failure self-management program designed for patients with low literacy versus usual care.

**Methods:**

We performed a 12-month randomized controlled trial. From November 2001 to April 2003, we enrolled participants aged 30–80, who had heart failure and took furosemide. Intervention patients received education on self-care emphasizing daily weight measurement, diuretic dose self-adjustment, and symptom recognition and response. Picture-based educational materials, a digital scale, and scheduled telephone follow-up were provided to reinforce adherence. Control patients received a generic heart failure brochure and usual care. Primary outcomes were combined hospitalization or death, and heart failure-related quality of life.

**Results:**

123 patients (64 control, 59 intervention) participated; 41% had inadequate literacy. Patients in the intervention group had a lower rate of hospitalization or death (crude incidence rate ratio (IRR) = 0.69; CI 0.4, 1.2; adjusted IRR = 0.53; CI 0.32, 0.89). This difference was larger for patients with low literacy (IRR = 0.39; CI 0.16, 0.91) than for higher literacy (IRR = 0.56; CI 0.3, 1.04), but the interaction was not statistically significant. At 12 months, more patients in the intervention group reported monitoring weights daily (79% vs. 29%, p < 0.0001). After adjusting for baseline demographic and treatment differences, we found no difference in heart failure-related quality of life at 12 months (difference = -2; CI -5, +9).

**Conclusion:**

A primary care-based heart failure self-management program designed for patients with low literacy reduces the risk of hospitalizations or death.

## Background

Limited literacy skills are common among adults in the United States [1]. Low literacy is associated with increased risk of hospitalization and worse control of chronic diseases [1-4]. Heart failure is a common chronic illness requiring multiple medications and significant self-care. Heart failure is the leading cause of hospitalization in the Medicare population [5]. The complexity of care for heart failure puts people with low literacy at considerable risk for adverse outcomes including hospitalization, worse quality of life, and mortality.

Heart failure disease-management interventions appear effective in reducing rehospitalizations and improving quality of life [6]. Most randomized clinical trials of heart failure disease management completed over the last 10 years have enrolled patients during, or shortly after, hospitalization and reported the outcome of readmission [6]. Although the designs of these programs vary, several have tested education and support to enhance patient self-management as the main component of the intervention [7-10]. The content of self-management education usually includes teaching to understand medications, reduce salt intake, monitor daily weights, and recognize symptoms. Most programs include structured follow-up either by home visit, phone, or mail. Only a few, uncontrolled studies specifically ask patients to self-adjust their diuretics [11,12].

Heart failure self-management programs may be particularly effective for vulnerable populations, such as those with poor literacy [13,14]. However, to our knowledge, no previous studies have explicitly examined the role of self-management programs in a low literacy population. A recently published study and accompanying editorial suggested that such self-management support may be most effective among vulnerable populations [13,14]. Low literacy may represent a vulnerability for which we should design our programs. Disease management for patients with low literacy may require refined approaches to foster self-management skills. We developed a heart failure self-management program for use by patients with a variety of literacy levels [15]. We performed a randomized controlled trial comparing our self-management program to usual care among outpatients to test if the program could reduce hospitalizations and improve heart failure-related quality of life.

## Methods

### Study design

We conducted a randomized controlled trial in the University of North Carolina (UNC) General Internal Medicine Practice, which serves a wide socioeconomic range of patients. The practice, staffed by over 20 attending faculty and 70 medical residents, cares for over 500 patients with heart failure.

### Study participants

To be eligible, patients had to have a clinical diagnosis of heart failure confirmed by their primary provider through a direct interview, and one of the following: 1) chest x-ray findings consistent with heart failure, 2) ejection fraction <40% by any method, or 3) a history of peripheral edema. They also had to have New York Heart Association class II-IV symptoms within the last 3 months. Patients were excluded if they had moderate to severe dementia (based on the treating physician's clinical judgment), terminal illness with life expectancy less than 6 months, severe hearing impairment, blindness, current substance abuse, a serum creatinine >4 mg/dl or on dialysis, a requirement of supplemental oxygen at home, lacked a telephone, or were scheduled to undergo cardiac surgery or awaiting heart transplant. We did not exclude patients on the basis of literacy skill because we felt patients of all literacy levels would benefit from this intervention. Patients who read well often prefer information presented in an easy-to-read format [16]. We accepted referrals from the cardiology clinic if patients met eligibility criteria. This study was approved by the Institutional Review Board of the UNC School of Medicine, and all participants gave informed consent prior to enrollment. For participants who could not adequately read the informed consent document, the research assistant read and explained it to them. They were asked to sign a short form indicating that the informed consent document was reviewed and they agreed to participate. When the short form was used, a witness was asked to attest to the adequacy of the consent process.

### Procedures

Participants were recruited between November 2001 and April 2003 from the General Internal Medicine and Cardiology Practices at UNC Hospitals. A trained research assistant screened all patients age 30–80 for use of furosemide. If the patient was on furosemide, their physician was queried about the presence of heart failure. If the patient met eligibility criteria and consented to participate, baseline data were collected. We then randomized patients by concealed allocation based on a random number generator to receive the intervention or usual care. All patients were followed for one year. All data were collected in the General Internal Medicine Practice.

### Intervention

The intervention was delivered in the General Internal Medicine Practice. The educational materials and disease management intervention were previously described in detail, and the intervention is summarized here [15].

The intervention began with a 1-hour educational session with a clinical pharmacist or health educator during a regular clinic visit. Patients were given an educational booklet designed for low literacy patients (written below the 6^th ^grade level and extensively pre-tested in focus groups and a pilot study [15]) and a digital scale. The educator and patient reviewed the booklet together, including management scenarios. As part of the educational session, patients were taught to identify signs of heart failure exacerbation, perform daily weight assessment, and adjust their diuretic dose. Because this intervention was aimed at patients with low literacy, the health educator used pedagogic strategies felt to improve comprehension for patients with low literacy [17]. For example, the educator had the patient teach back the information [18], engaged the patient in filling out the notebook, and used brainstorming to help the patient incorporate self-management into their lives.

**Figure 1:**
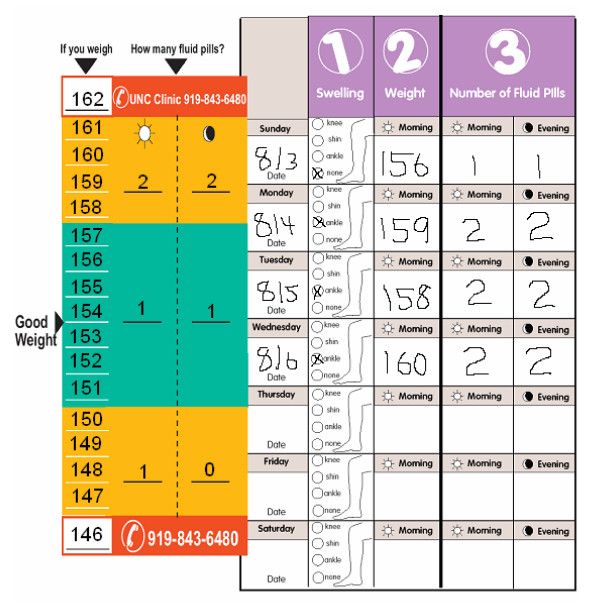
Sample heart failure management plan for a patient.

The educator, patient, and primary care physician collaborated to establish the patient's "good weight" (i.e., where the patient's heart failure was stable) and baseline diuretic dose. The educator then filled in the management plan in the patient's notebook to help the patient better manage weight fluctuations and self-adjust the diuretic dose based on weight (Figure 1). The general plan involved doubling the dosage if weight went up and halving it if weight went down.

The program coordinator then made scheduled follow-up phone calls (days 3, 7, 14, 21, 28, 56) and monthly during months 3–6. The follow-up phone calls, each lasting 5–15 minutes, were designed to reinforce the educational session and provide motivation for the patients. Again, the program coordinator had the patient describe their self-management practices and offered feedback to improve them. Patients experiencing worsening symptoms were scheduled acute visits with their physician. We did not provide specialized nursing assessment, care or medication advice beyond diuretic dosing. If the patient's doctor determined that the good weight had changed, the program coordinator would revise the care plan with the patient.

Patients enrolled in the control group received a general heart failure education pamphlet written at approximately the 7^th ^grade level, and continued with usual care from their primary physician. The only contacts between the research team and the control patients were at enrollment and data collection.

### Measures

We assessed outcomes at 6 and 12 months through in-person interviews and review of the medical record. To be sensitive to low literacy, all interviews were conducted verbally by a trained research assistant. If patients were unable to come to clinic for the interview, it was conducted by phone. The research assistant was not blinded to the patient's study group.

Primary outcomes were death or all-cause readmission and heart failure-related quality of life at the end of 12 months. Data on hospitalization dates were obtained from the patient and confirmed by medical chart review. All admissions, regardless of hospital or cause, were counted.

For exploratory analyses, we classified reason for admission as cardiac or non-cardiac. Cardiac admissions included those primarily for heart failure (e.g., shortness of breath and edema relieved by diuresis) and other cardiac causes such as chest pain, arrhythmias, or syncope. Cause of admission was determined by chart review by one of the authors (D.D.) who was blinded to treatment allocation.

Heart failure-related quality of life was assessed using a modified version of the Minnesota Living with Heart Failure Questionnaire (MLHF). The MLHF is a 21 question instrument with a 6-point Likert scale response option and scores ranging from 0 to 105 [19]. In pilot testing of the MLHF, we found that low literacy patients had trouble answering questions with the standard 6-point Likert scale [15], so we changed the response scale to 4 points, using a visual display with stars to represent increasing severity. The 4-point Likert scale was scored as 0, 1, 3, and 5 to approximate standard scores on the MLHF.

Secondary measures included heart failure self-efficacy, knowledge, and behaviors. Self-efficacy was measured with an 8 item scale developed for the behaviors needed in this trial as suggested by self-efficacy scale developers [20]. Respondents used a 4-point Likert scale yielding a total score from 0–24. We assessed heart failure knowledge using a knowledge test previously developed for this population [15], Heart failure self-management behavior was assessed by asking patients how often they weighed themselves.

We used patient self-report and the medical chart to measure age, gender, race, insurance status, income, years of education, medication use, years with heart failure, and the presence of co-morbidities. We measured literacy using the Short Test of Functional Health Literacy in Adults (S-TOFHLA) [21], a well-validated scale that correlates well with other measures of reading ability [22]. Patients who scored in the inadequate literacy range on the S-TOFHLA were considered to have "low literacy." The cut-point for inadequate literacy is roughly analogous to the 4^th ^grade reading level.

### Sample size

Sample size was based on pilot study results showing a 9-point improvement in MLHF scores over 3-months with the intervention [15]. Detecting a 9-point difference between intervention and control group with 80% power and alpha set at 0.05 required 70 patients per group. We aimed to enroll 150 patients to account for possible attrition, but stopped at 127 because funding ended. We did not power this study to detect differences in hospitalization, but studies with even smaller numbers of patients have shown a difference for that outcome [7].

### Statistical methods

Patients who did not return any phone calls and did not return for follow-up assessment did not have outcome data for analysis. Patients who withdrew from the study were censored at the time of withdrawal; any data collected prior to withdrawal were included in the analysis. Baseline differences between groups were assessed using t-tests for continuous outcomes and chi-squared tests for categorical outcomes.

For MLHF, heart failure self-efficacy and heart failure knowledge, we used two-sample t-tests. Non-parametric tests were also performed for all comparisons, but results did not differ from the parametric tests. Because of the small sample size and the unequal distribution of baseline characteristics, we adjusted for baseline differences using linear regression. Analyses of self-reported outcomes, such as MLHF, excluded patients who died or withdrew from the study before 6 or 12 month data was collected.

For hospitalization or death, we used negative binomial regression and censored patients at the time of death or withdrawal from the study. Based on the likelihood ratio test, negative binomial regression was a better fit for the data than a Poisson regression. Additionally, the Vuong test confirmed that a zero-inflated model was inappropriate [23].

Because of uneven distribution of baseline characteristics, we performed analysis of covariance with negative binomial regression to control for baseline differences [24]. We identified the following variables that could contribute to hospitalization or death based on previous studies: age, race, gender, literacy level, hypertension, diabetes, creatinine, MLHF score, use of β-blockers, angiotensin converting enzyme (ACE) inhibitors or angiotensin receptor blockers (ARBs), use of digoxin, and presence of systolic dysfunction [7,25]. Variables were not included in the model if the relationship between the variable and the study group or outcome had a p value greater than 0.3. We started with a model including the following items to arrive at the best point estimate: age, gender, hypertension, creatinine, MLHF, use of β-blockers, and use of ACE inhibitors or ARBs. We then eliminated variables from the model if p > 0.30, and if the point estimate remained within 10% of the initial estimate.

We prespecified a subgroup analysis in patients with low literacy to analyze if the intervention had a similar effect. The same analysis described above was repeated for the subgroups of patients with low literacy and those with higher literacy. The initial multivariate model for the subgroups analysis included: age, gender, hypertension, MLHF, use of β-blockers, and use of ACE inhibitors or ARBs.

### Role of the funding source

The funding sources had no role in the design and conduct of the study; collection, management, analysis, and interpretation of the data; or preparation, review, or approval of the manuscript.

## Results

### Patients

**Figure 2:**
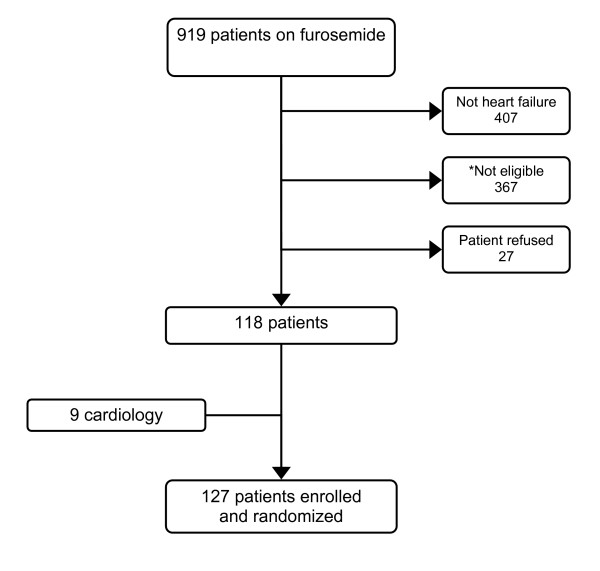
Screening and enrollment results.

We screened 919 patients on furosemide between November 2001 and April 2003. 127 met eligibility criteria and agreed to participate (Figure 2). Of those not enrolled, 407 did not have heart failure according to their physician, 367 did not meet eligibility criteria and 27 declined to participate.

**Figure 3:**
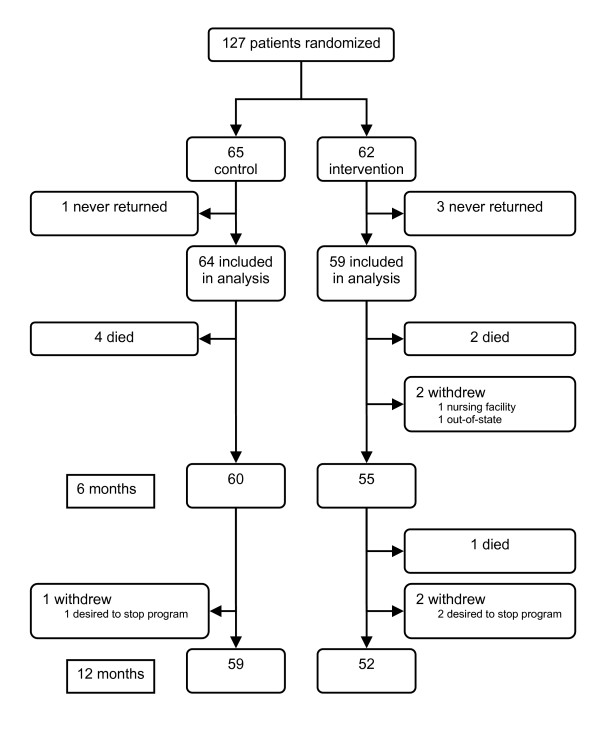
Patient flow during the study.

Of those randomized to the control group, 1 never returned after the first visit, 1 withdrew during the study and 4 died during the study. Follow-up was completed for all of the remaining participants (98%) (Figure 3). Of those randomized to the intervention group, 3 never returned after the first visit, 4 withdrew during the study and 3 died during the study. Follow-up was completed for all of the remaining participants (93%).

At baseline, most characteristics were similar between the two groups (Table 1). However, the control group had more participants with hypertension, fewer with diabetes, and fewer men. Of heart failure related characteristics, the control group had more participants with systolic dysfunction, and taking β-blockers. The intervention group had more participants taking ACE inhibitors or ARBs, and digoxin. Regardless of these differences, none were statistically significant. The control group did have statistically significantly higher baseline MLHF scores representing worse symptoms at baseline.

### Hospitalization or death

There were 68 hospitalizations (65) or deaths (3) in the intervention group and 111 (107 hospitalizations, 4 deaths) in the control group. The crude all-cause hospital admission or death incidence rate ratio (IRR) was 0.69 (95% CI 0.40, 1.19). After adjusting for age, gender, use of ACE inhibitor or ARB, use of a β-blocker, presence of hypertension, and baseline MLHF, intervention patients were less likely to have the outcome (IRR = 0.53; 95% CI 0.32, 0.89). 61% of patients in the control group had at least one hospitalization or died, and 42% of patients in the intervention group had at least 1 hospitalization or died (p = 0.13).

### Cardiac hospitalization

39% of patients in the control group and 34% of patients in the intervention group had at least one hospitalization for cardiac causes (p = 0.55). The unadjusted IRR was 0.79 (95% CI 0.42, 1.5). After adjusting for baseline differences, the IRR was 0.85 (95% CI 0.44, 1.7).

### Heart failure-related quality of life

In unadjusted analysis, the control group, on average, improved 5 points on the MLHF and the intervention group improved by 1 point. The difference was not statistically significant (3.5 points, 95% CI 11, -4, p = 0.36). After adjusting for baseline differences between the groups, the difference was 2 points (95% CI 9, -5, p = 0.59) suggesting no effect on heart failure-related quality of life.

### Other outcomes

#### Knowledge

Heart failure related knowledge improved more in the intervention group than in the control group. Mean difference in score improvement was 12 percentage points (95% CI 6, 18; p < 0.001).

#### Self-efficacy

Heart failure self-efficacy improved more in the intervention group than in the control group. Mean difference in score improvement was 2 points (95% CI 0.7, 3.1; p = 0.0026).

#### Self-care behavior

Significantly more patients in the intervention group than in the control group reported daily weight measurement at 12 months (79% vs. 29%, p < 0.001).

### Subgroup analyses according to literacy

Twenty-four patients in each group had inadequate literacy based on the S-TOFHLA (Table 2). Among these patients, there was no difference in quality of life score in unadjusted and adjusted analyses (difference = -1.6; 95% CI -15, 12); p = 0.81). For the combined outcome of hospitalization or death, the unadjusted IRR was 0.77 (95% CI 0.30, 1.94). After adjusting for baseline differences, the IRR was 0.39 (95% CI 0.16, 0.91).

Seventy-five patients had marginal or adequate literacy based on the S-TOFHLA. We found no difference in quality of life score in unadjusted and adjusted analyses (difference = -4.2; 95% CI -14, 6; p = 0.40). Among patients in the higher literacy group, the unadjusted IRR for hospitalization or death was 0.65 (95% CI 0.33, 1.3). After adjusting for baseline differences, the IRR was 0.56 (95% CI 0.30, 1.04). We did not find a statistically significant effect modification between literacy and the intervention.

## Discussion

A heart failure self-management program designed for patients with low literacy reduced the rate of the combined endpoint of hospitalization or death. The prespecified subgroup analyses suggest that patients with low literacy benefited as much from the intervention as the patients with higher literacy. The success of our intervention reflects the goals of our program. We designed an easy-to-read and use educational booklet and self-management plan, and focused on overcoming barriers to learning self-management [15].

Our intervention was founded on teaching self-management. We focused on helping patients understand signs and symptoms of worsening heart failure and perform self-adjustment of diuretics based on weight fluctuation. Many care providers would not attempt to teach patients, particularly those with low literacy, how to self-adjust their diuretic medication. We found that, with careful teaching, many patients incorporated this strategy into their daily routine successfully, as demonstrated by improved self-care behaviors. Teaching self-adjustment of diuretics, rather than the conventional teaching to call the care provider if weight fluctuates, empowers patients to take more control over their illness.

Self-adjustment of diuretic dose is a prominent aspect of the self-management training we provided to the intervention patients. Other programs to improve patient self-management have not been explicit in teaching patients to self-adjust their diuretic dose based on weight fluctuation. Although our outcomes are comparable to others', using this approach puts more control into the hands of the patient. Furthermore, our intervention appears effective among patients with low literacy skills, a group often overlooked for empowering interventions.

Our study adds to the growing literature on disease management programs for patients with heart failure [6], particularly those that focus on self-management training [7-10]. Studies focusing on self-management training have demonstrated comparable improvements in hospitalization rates to more comprehensive programs that aim to improve the quality of pharmaceutical prescribing, provide home visits, and take place in specialized cardiology clinics [6]. Such comprehensive programs have also been shown to reduce mortality, but self-management programs have not [6].

We did not detect any difference in heart failure related quality of life which was the outcome we powered our study to detect. Other self-management studies that have found improved quality of life have enrolled patients during a heart failure hospitalization [8,9]; however, we enrolled patients in the outpatient setting while they were clinically stable. Improving quality of life for stable outpatients may be more difficult for this type of intervention.

We have previously reported that patients with diabetes and low literacy benefited more from a disease management intervention than those with higher literacy skills [26]. A similar result in two different chronic diseases substantiates the claim that appropriately designed disease management programs may have greater effect for low literacy or vulnerable populations, who are most at risk for adverse outcomes with usual care.

This finding is particularly important in light of the recent study by DeBusk and colleagues that did not find a reduction in hospitalization with a well-designed comprehensive intervention [13]. The authors and an accompanying editorial [14] suggested that the failure to detect improvement may have occurred because the patients studied were less at-risk than in other studies. They called for more research to determine better ways of targeting disease management. We believe that low literacy is an important marker for vulnerability to adverse outcomes, and that disease management programs targeted to patients with low literacy may be an effective way of focusing resources on those most able to benefit. If patients with low literacy are to be preferentially recruited for such programs, innovative outreach and screening efforts will likely be required, as patients with low literacy may face particular barriers to accessing such care.

This study should be interpreted in light of its limitations. Research assistants were not blind to group assignment during the assessment of self-reported outcomes. As such, patients in the intervention may have been more likely to inflate their responses in an effort to please the interviewer. This effect would tend to inflate patient responses to the subjective assessments of heart failure-related quality of life, self-efficacy, and self-care behaviors. The MLHF questionnaire was modified from its original form to make it easier for patients with low literacy to respond. This change in the scale may have changed its ability to detect important changes in heart failure related quality of life. Because the groups' mean scores were almost identical, we do not feel this limitation changed our results. In a similar vein, most questionnaires are not validated in low literacy populations, raising questions as to their ability to perform to the same standards.

Our sample size was small, which did not allow for an even distribution of baseline variables among the groups. We controlled for baseline differences between groups in our analysis. While it is controversial whether or not to control for baseline differences in randomized controlled trials, some analysts have argued that doing so improves the power without introducing bias [24]. A larger, multi-site study would offer better control of confounders, better generalizability, and more power to determine differences in effect according to literacy.

We did not collect data on the resources needed to implement this type of intervention in usual settings, and such a study and cost-effectiveness analysis would be helpful for most interventions of this type. We used health educators, not nurses or physicians, to deliver the intervention. By using less highly trained individuals to deliver the intervention, we enabled nurses and physicians to perform clinical tasks more commensurate with their training. Other studies that have performed global cost estimates have found that the savings from reductions in hospitalizations exceed the cost of the intervention [6].

## Conclusion

In conclusion, our heart failure self-management program, designed for patients of all literacy levels, appears to reduce rates of hospitalization and death. Patients with low literacy, and other vulnerable patients, may stand to benefit most from these programs. Further research into the design, implementation, and dissemination of disease management programs for low literacy patients will be crucial for meeting the health care needs of the growing population of patients with chronic illness.

## Competing interests

Drs. DeWalt and Pignone have received honoraria and grants from Pfizer, Inc., Dr. Rothman has received grants from Pfizer, Inc., and Dr. Sueta is in their speakers bureau.

## Authors' contributions

DD conceived of the study, participated in its design and coordination, performed statistical analyses, interpretation of the data, and drafted the manuscript. RM, MB conceived of the study and participated in its coordination. MK, KC coordinated the study, and collected the data, RR, CS participated in study design and interpretation of the data. MP conceived of the study, participated in its design and coordination, and interpretation of the data. All authors reviewed the manuscript for important intellectual content and gave final approval.

## Pre-publication history

The pre-publication history for this paper can be accessed here:



## Figures and Tables

**Table 1:** Baseline characteristics.

Variable	Control (n = 64)	Intervention (n = 59)	P value
Mean Age, years (SD)	62 (11)	63 (9)	0.37
African American, %	55	54	0.96
Male, %	41	58	0.059
Education, years (SD)	9.9 (2.6)	9.1 (3.2)	0.17
Income <15,000/yr, %	67	69	0.60
Medicaid, %	33	34	0.90
Medicare, %	72	71	0.61
Inadequate Literacy, %	39	42	0.43
Hypertension, %	89	86	0.66
Diabetes, %	52	59	0.39
Creatinine, mg/dl	1.3	1.2	0.19
Time with heart failure, mean years (SD)	7 (8)	6 (9)	0.75
NYHA class, %			0.66
II	47	53	
III	51	40	
IV	2	7	
Systolic dysfunction, %	44	39	0.73
Medications, %			
ACEI or ARB	69	78	0.25
β-blocker	72	54	0.04
Digoxin	19	25	0.39
Knowledge, mean percent	57	55	0.67
Self-efficacy, mean score	22	22	0.64
Daily weight measurement, %	15	13	0.64
HFQOL score, mean score (range 0–105)	57	45	0.0028

NYHA: New York Heart Association; ACEI: Angiotensin Converting Enzyme Inhibitor; HFQOL: Heart Failure-Related Quality of Life

**Table 2:** Baseline characteristics for the subgroup with low literacy.

Variable	Control (n = 24)	Intervention (n = 24)	P value
Mean Age, years (SD)	65 (10)	68 (8)	0.38
African American, %	71	71	1.0
Male, %	46	71	0.079
Education, years (SD)	8.6 (2.9)	6.3 (2.9)	0.009
Income <15,000/yr, %	83	88	0.33
Medicaid, %	45	45	1.0
Medicare, %	75	79	0.73
Inadequate Literacy, %	39	42	0.43
Hypertension, %	92	83	0.38
Diabetes, %	63	71	0.54
Creatinine, mg/dl	1.4	1.3	0.43
Time with heart failure, mean years (SD)	7 (7)	8 (11)	0.63
NYHA class, %			0.55
II	58	50	
III	42	46	
IV	0	4	
Systolic dysfunction, %	38	42	0.55
Medications, %			
ACEI or ARB, %	83	79	0.14
b-blocker, %	71	42	0.04
Digoxin, %	17	21	0.39
Knowledge, mean percent	46	49	0.40
Self-efficacy, mean score	21	21	0.78
Daily weight measurement, %	17	4	0.16
HFQOL score, mean score (range 0–105)	51.9	42	0.11

NYHA: New York Heart Association; ACEI: Angiotensin Converting Enzyme Inhibitor; HFQOL: Heart Failure-Related Quality of Life
